# *Vipera berus berus* Venom from Russia: Venomics, Bioactivities and Preclinical Assessment of Microgen Antivenom

**DOI:** 10.3390/toxins11020090

**Published:** 2019-02-01

**Authors:** Ruslan I. Al-Shekhadat, Ksenia S. Lopushanskaya, Álvaro Segura, José María Gutiérrez, Juan J. Calvete, Davinia Pla

**Affiliations:** 1 LLC Innova Plus, Saint-Petersburg 198099, Russia; ghevachka@mail.ru; 2 Instituto Clodomiro Picado, Facultad de Microbiología, Universidad de Costa Rica, San José 11501-206, Costa Rica; alvaro.seguraruiz@ucr.ac.cr (Á.S.); jose.gutierrez@ucr.ac.cr (J.M.G.); 3 Laboratorio de Venómica Evolutiva y Traslacional, CSIC, Jaime Roig 11, 46010 Valencia, Spain

**Keywords:** snake venom, common European viper, *Vipera berus berus*, venomics, snake antivenom, antivenomics

## Abstract

The common European adder, *Vipera berus berus*, is a medically relevant species, which is widely distributed in Russia and thus, is responsible for most snakebite accidents in Russia. We have investigated the toxic and enzymatic activities and have determined the proteomic composition of its venom. Phospholipases A_2_ (PLA_2_, 25.3% of the venom proteome), serine proteinases (SVSP, 16.2%), metalloproteinases (SVMP, 17.2%), vasoactive peptides (bradykinin-potentiating peptides (BPPs), 9.5% and C-type natriuretic peptides (C-NAP, 7.8%), cysteine-rich secretory protein (CRISP, 8%) and L-amino acid oxidase (LAO, 7.3%) represent the major toxin classes found in *V. b. berus* (Russia) venom. This study was also designed to assess the in vivo and in vitro preclinical efficacy of the Russian Microgen antivenom in neutralizing the main effects of *V. b. berus* venom. The results show that this antivenom is capable of neutralizing the lethal, hemorrhagic and PLA_2_ activities. Third-generation antivenomics was applied to quantify the toxin-recognition landscape and the maximal binding capacity of the antivenom for each component of the venom. The antivenomics analysis revealed that 6.24% of the anti-*V. b. berus* F(ab’)_2_ molecules fraction are toxin-binding antibodies, 60% of which represent clinically relevant antivenom molecules.

## 1. Introduction

Old World true vipers are comprised of three major clades whose origin stem from a basal segregation of the *Vipera sensu lato* group on three landmasses during the early Miocene period (23.3–16.3 million years ago). These landmasses were namely Europe, the Middle East and North Africa, which were separated by the Mediterranean and Paratethys Seas [1,2,3]. The systematics of *Vipera s. l.* has always been problematic and is constantly being revised [4,5,6,7,8]. Three clades, including 24 species of extant Eurasian snakes, the *V. aspis* group, the *V. ammodytes* complex and the *Pelias* group, are recognized within the monophyletic *Vipera* (Laurenti 1768) genus [1,2,3,9,10].

The *Pelias* group, which comprises the most primitive representatives of the genus, colonized Northern Europe, whereas *V. aspis* and *V. ammodytes* migrated towards Southern Europe. *Pelias* is subclassified into two subgroups. One was comprised of *V. dinniki*, *V. kasnakovi* and *V. ursinii* while another included *V. berus*, *V. barani*, *V. nikolskii* and *V. seoanei*. The common European viper (or adder), *Vipera berus* (Linnaeus 1758) [11], has the widest distribution of any other terrestrial snake. It can be found in a variety of complex habitats from northwestern Europe (Scotland, 6°W), eastwards across Europe and central Russian, northern Mongolia, China and Korea to Sakhalin Island on the Pacific coast (143°E). Furthermore, it can also be found from its most southerly distribution in the Balkans (42°N) to north of the Arctic Circle (Fennoscandia, 69°N) [4,5,6]. Despite of its vast distribution and enormous range of variability amongst populations [12,13,14,15], the taxon *Vipera berus* is arranged in only four subspecies [4], which are namely *V. b. berus* (Linnaeus 1758), the Balkan adder *V. b. bosniensis* (Boettger 1889), *V. b. nikolskii* (Vedmederya, Grubant & Rudajewa 1986) and *V. b. sachalinensis*, which are distinguished both by lepidotic and coloration traits [5]. Analysis of the genetic structure and colonization history of *V. berus* across the entire distribution range [16] has revealed three major mitochondrial lineages, which originated during the Lower-Mid Pleistocene (about 1.4 million years ago, Mya) from an Italian, a Balkan and a Northern (from France to Russia) interglacial refugial areas in Eastern Europe near the Carpathian Mountains. The Northern clade presents an important substructure attributed to two sequential colonization events in Europe during the last glacial cycles, which occurred in the Mid–Late Pleistocene (dated at 0.7 Mya; source of the Eastern Europe to Pacific Russia eastern subclade) and 0.21 Mya (Western clade; the origin of the refugial population located west of the Alps) [16,17,18]. This evidence suggests that present-day genetic and morphological differentiation between *V. berus* lineages are related to recent local adaptations and some authors recognize *V. b. bosniensis* and *V. b. sachalinensis* as valid species [6,9].

The common European adder is a relatively thick-bodied small viper with an average adult size of 60–70 cm. Although it is not considered to be aggressive and usually bites only when provoked, stepped on, or picked up [4,5,6], *V. berus* causes more bites than any of its congeners [19,20]. A *V. b. berus* bite can inject about 10–18 mg of venom, with the median lethal dose (LD_50_) for mice being 0.55 mg/kg IV, 0.80 mg/kg IP and 6.45 mg/kg SC [21]. Bites can be very painful, but are seldom fatal [22]. The local effects of *V. b. berus* bites include hemorrhage, edema, myonecrosis and bruising. The most common signs of systemic envenoming are typically anaphylactic-like symptoms, such as nausea, vomiting, diarrhea and gastrointestinal symptoms. Other systemic effects can include abdominal colic, incontinence, sweating, vasoconstriction, tachycardia, angio-edema of the face, lips, gums, tongue, throat and epiglottis, urticaria and bronchospasm [22,23,24]. Reports of neurotoxic effects [24,25,26,27], systemic hemorrhage and coagulopathy following *V. b. berus* envenoming are rare [20,22]. However, neurotoxic activity is an intrinsic part of the venom of the Balkan adder (*V. b. bosniensis*) [28].

A retrospective study spanning the period of 1980–2010 revealed that the incidence of severe (grades II and III) adder bites in Europe was 0.6 per million population per year, with a peak in the summer month [19]. Antivenom was used in 35% of the cases. The prevalence of undesirable side-effects following antivenom administration was 4% and the annual number of deaths was less than 5 [19]. The study also revealed a significant fall in mortality in most European countries during the 20th century. In Sweden, where the longest and most complete series has been recorded, the average annual mortality per 100,000 population fell from 0.02 (1911–1949) [29] to 0.003 (1950–1979) [30] and to 0.0008 per 100,000 (1980–2009) [19]. This decrease in mortality has been attributed to the development of reanimation and other intensive care interventions in addition to the use of highly-purified antivenoms since 1995 [23].

Of the seven antivenoms available for European *Vipera* spp. envenoming, four declare that they have neutralization efficacy against *V. berus* venom and WHO only recommends that two antivenoms, ViperaTAb (Micropharm, UK) [31,32] and ViperFAV (Sanofi-Pasteur, France) [33], should be administered by the intravenous route [34]. Other antivenoms are usually given by the intramuscular route and lack any evidence of effectiveness. There is a lack of information on the preclinical efficacy of another antivenom, Anti-Viper Venom Serum, manufactured by the Federal State Company for Immunobiological Medicines, Microgen (Moscow, Russia), despite it being registered in the Russian Federation for *V. berus* envenoming. This study was designed to assess the preclinical efficacy of the Russian Anti-Viper Venom antivenom to neutralize three key effects of *V. b. berus* venom, i.e., lethal, hemorrhagic and phospholipase A_2_ activity, by combination of in vivo neutralization assays [35] and in vitro third-generation antivenomics analysis [36,37]. This will ultimately help in assessing the toxin recognition landscape of the antivenom and quantify the fraction of therapeutic antivenom molecules.

## 2. Results and Discussion

### 2.1. Toxic and Enzymatic Activities of V. b. berus Venom and their Neutralization by Microgen Antivenom

Table 1 shows the results of the quantification of lethal, hemorrhagic and PLA_2_ activities of the venom of *V. b. berus* (Russia) and the results of the neutralizing ability of the monospecific Microgen antivenom against these activities in mice. In agreement with previous studies [38], the venom exerted lethal, hemorrhagic, and PLA_2_ effects. The antivenom was effective in the neutralization of these venom activities (Table 1).

The lethality neutralization potency of the antivenom (P, the amount of venom neutralized by the antivenom) was calculated as follows [39]:

P = [(*n*−1)/mL AV/mouse] × LD_50_ [mg V/mouse], (1)

where *n* = number of LD_50_ in the test dose and = [(4−1)/0.0258] × 0.0198 = 2.3 mg venom neutralized per mL antivenom.

This figure indicates that 1 mL of Microgen antivenom has the capability to completely prevent the mortality of all tested mice when they were given 116 LD_50_s of *V. b. berus* venom and thus, fulfils the pharmacopoeial minimum specification of not less than 50 LD_50_s/mL.

### 2.2. Quantification of Venom-Specific Antivenom Antibodies using Third-Generation Antivenomics

Antivenomics is a translational venomics approach designed to complement in vivo preclinical neutralization assays. It provides qualitative and quantitative information on the binding capacity of an antivenom toward the different toxins present in a venom and on the fraction of venom-specific antibodies present in a given antivenom [36]. To achieve locus resolution in antivenomics analysis, a *sine qua non* condition is to know the composition of the venom proteome [37]. To fulfill this prerequisite, the venom of *V. b. berus* (Russia) was subjected to venomics characterization [40,41].

#### 2.2.1. Characterization of the V. b. berus Venom Proteome

**Figure 1:**
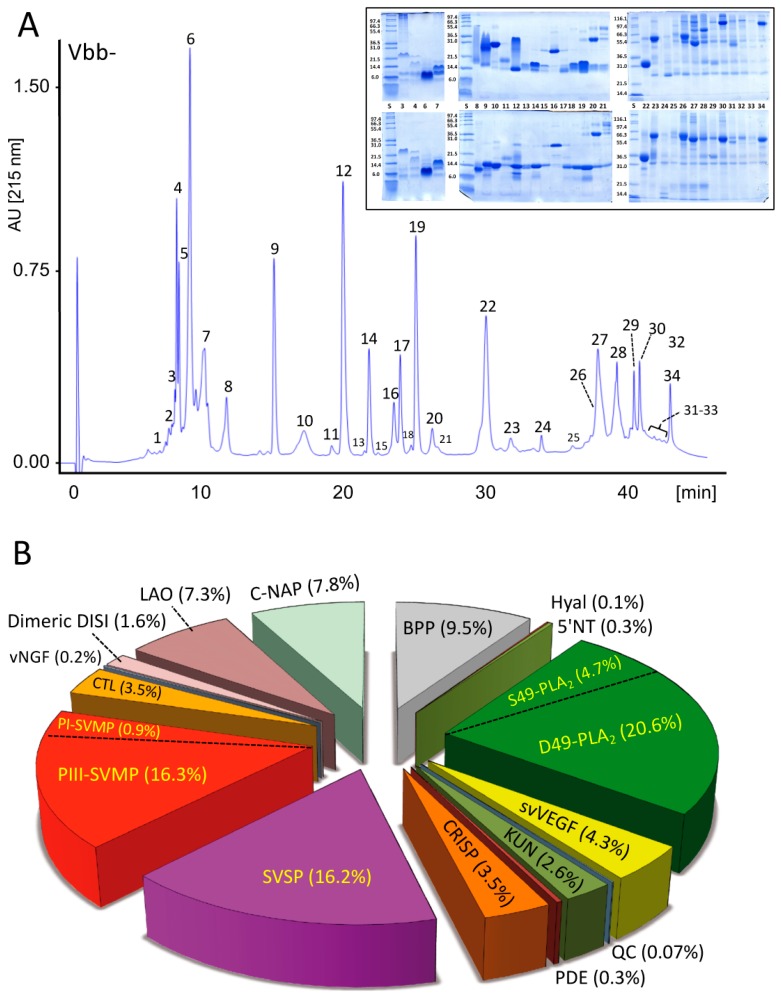
Proteomic analyses of venom of *V. b. berus* from Russia. (**A**) Reverse-phase chromatographic separation of the venom proteins. Inset, SDS–PAGE profile of the chromatographic fractions analyzed under non-reduced (upper gels) and reduced (lower gels) conditions. (**B**) Pie chart displaying the relative occurrence (in percentage of total venom proteins) of toxins from different protein families in the venom proteome. BPP, Bradykinin potentiating peptides; Hyal, hyaluronidase; 5′NT, 5′-nucleotidase; S39 and D49 PLA_2_, phosphilipases A_2_ containing serine and aspartic acid residues in amino acid sequence position 39, respectively; svVEGF, snake venom vascular endothelial growth factor; QC, glutaminyl-peptide cyclotransferase; KUN, Kunitz-type proteinase inhibitor; PDE, phosphodiesterase; CRISP, cysteine-rich secretory protein; SVSP, snake venom serine proteinase; PI- and PIII-SVMP, metalloproteinases of class PI and PIII, respectively; CTL, C-type lectin-like protein; vNGF, venom nerve growth factor; DISI, disintegrin; LAO, L-amino acid oxidase; C-NAP, C-type natriuretic peptide.

The venom proteome of *V. b. berus* (Russia) was characterized and quantified by applying the previously described [40,41] two-step pre-MS decomplexation protocol (reverse-phase HPLC and SDS-PAGE) (Figure 1, panels A and B), followed by peptide-centric bottom-up analysis of tryptic digests from SDS-PAGE separated protein bands eluted in the different reverse-phase chromatographic fractions (Figure 1, inset in panel A). Supplementary Table S1 provides the details of the MS/MS assignment, quantification and database matching of the SDS-PAGE separated protein bands eluted in the 34 reverse-phase chromatographic fractions. Russian *V. b. berus* venom proteome comprises a complex toxin arsenal constituted by >80 distinct proteins of 15 toxin classes (Figure 1B). This is dominated by at least 18 D49-PLA_2_s and a single S49-PLA_2_, which comprise 20.6% and 4.7% of the venom proteome, respectively (Table S1). Other major toxin families are SVMPs (17.2%), SVSPs (16.2%), BPPs (9.5%), CRISP (8%), C-NAP (7.8%) and LAO (7.3%). This venom composition significantly departs from those reported for a pooled venom of European adders captured in the Slovak Republic at altitudes ranging from 650 to 750 m above sea level [42], but also from Vbb venom obtained from the Serpentarium of the Central Trade Base ‘Zoo-obyedinenie’ Khimky (Moscow region, Russia) [43]. Both venom proteomes were characterized by two-dimensional electrophoresis (2DE) followed by MS/MS analysis. The Slovakian venom is comprised of 25 proteins and peptides from 7 toxin families, with PLA_2_s representing the most abundant (59%) components [41]. The Russian venom [43] was resolved into 160 distinct 2DE spots and were assigned to 31 distinct proteins belonging to 11 toxin classes, with the predominance of SVSP (31%), SVMP (19%), C-NAP (11%), PLA_2_ (10%), CRISP (8%) and a smaller representation of SVMP inhibitor (4%), C-type lectin-like (CTL) and LAO (2%), Disintegrin (1%), aspartic protease (0.1%) and Kunitz-type proteinase inhibitors (KUN, 0.07%). These compositional differences may be due to geographical variations in the source of venoms and the different methodological strategies followed in the employed venomics approaches [44].

#### 2.2.2. Third-Generation Antivenomics

**Figure 2:**
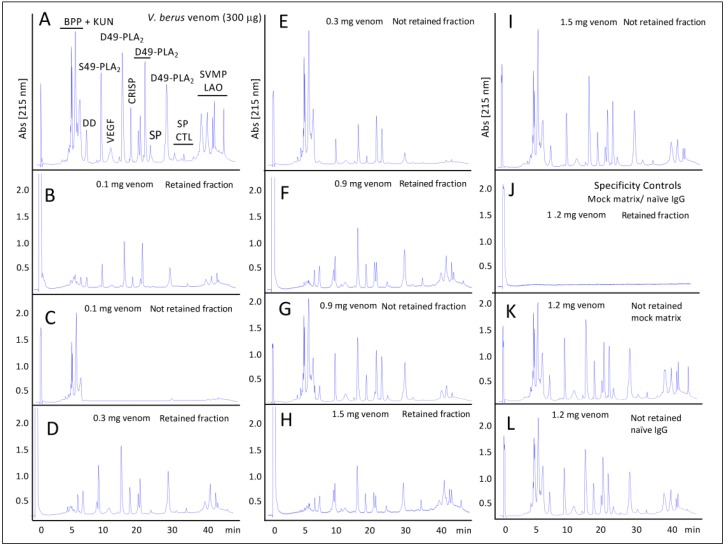
Third-Generation Antivenomics. Panel **A**, reverse-phase HPLC profile of 300 µg of *V. b. berus* (Russia) venom, highlighting the identification of major proteins eluted in the various chromatographic peaks (acronyms as in Figure 1) Panels **B** to **I** correspond to the non-immunoretained and immunoretained fractions recovered from the immunoaffinity columns after incubation with varying amounts (0.1–1.5 mg) of venom. Panels J to L: Specificity controls with mock chromatographic matrix and naïve horse IgGs.

The immunological reactivity of the therapeutic antivenom manufactured by “Microgen” LTD, Russia, was assessed by third-generation immunoaffinity-based antivenomics for the treatment of *V. b. berus* envenoming [36], as described for other antivenoms [45,46]. Figure 2 displays the series of antivenomics experiments in which a fixed amount (10 mg) of antivenom was confronted with the increasing amounts of *V. b. berus* venom. Table 2 shows the venom concentration-dependent maximal binding of the different RP-HPLC fraction to the affinity columns.

With the exception of the first three peaks that are comprised of small peptides, the rest of the fractions were retained in the immunoaffinity column incubated with the lowest amount (100 µg) of venom although these were to a varied extent. The pathophysiological relevance of these poorly immunogenic peptides has not been addressed in the current study. However, intraperitoneal administration of similar early-eluting peptide fractions from *Lachesis* venoms did not induce a significant change in the mean arterial blood pressure of mice, signs of abnormal behavior or histopathological alterations [47]. At higher venom concentrations, all the other venom fractions showed partitions between the non-retained and retained chromatographic fractions, which reached maximum binding at concentrations between 600–1200 µg of incubated venom.

From the antivenomics results shown in Table 2, it was calculated that 10 mg of immobilized antivenom F(ab’)_2_ fragments had a maximal binding capacity of 317.74 µg *V. b. berus* total venom proteins (31.77 mg venom/g F(ab’)_2_). The total amount of venom proteins bound per vial (1 mL, 120 mg F(ab’)_2_/mL) was 3.82 mg. Considering a nominal average molecular mass for the *V. b. berus* toxins of 28 kDa (calculated as ∑ (% i × M_i_), where % i is the relative abundance of toxin “i” and M_i_ is its molecular mass in Da), this amount of venom equals 0.136 µM of venom molecules. Assuming that the two antigen binding sites of an F(ab’)_2_ molecule were occupied at maximal antigen binding capacity, an anti-*V. b. berus* vial contained (0.136/2 = 0.068) µM of toxin-binding molecules or 7.49 mg F(ab’)_2_ (molar mass, 110 g/mol). This figure corresponds to 6.24% [(7.49/120) × 100] of the total anti-*V. b. berus* F(ab’)_2_ molecules. On the other hand, the fraction of toxin-binding anti-*V. b. berus* F(ab’)_2_ molecules that contributed to protect the test animals from the venom’s effect was calculated by dividing the antivenom’s potency (P, 2.3 mg V/mL AV) by its maximal capacity to bind total venom proteins (3.82 mg V/mL AV):

% toxin-binding and neutralizing anti-*V. b. berus* F(ab’)_2_ molecules = [2.3/3.82] × 100 = 60.2% (2)

By combining this figure with the above antivenomics-derived percentage of anti-*V. b. berus* F(ab’)_2_ molecules bearing affinity towards *V. b. berus* venom toxins (6.24%), we determined that [(60.2 × 6.24)/100] = 3.76% of the antivenom F(ab’)_2_ antibodies corresponded to clinically relevant antivenom molecules. This figure clearly suggests that there is enough room to improve the immunization procedure to increase the relative amount of anti-toxin antibodies, which ranges between 15–30% of the total antivenom antibodies in other antivenoms [45,48].

The preclinical parameters provided in our study represent a proxy for the nominal efficacy limits of the antivenom. However, translating this information into an accurate calculation of the number of vials needed to treat a bite is not a trivial matter. Reversing the symptoms of an envenoming is synonymous with shifting the balance between the amount of circulating and target-bound venom toxins, with toxin–antibody complexes contributing to the latter. This requires maintaining an a priori unknown concentration of circulating antivenom antibodies and the clearance of the antigen–antibody complexes. The effective antivenom concentration needed to clear the venom toxins from the envenomed body below an also a priori unknown threshold will depend on the pharmacokinetics of the antivenom antibodies and the affinity of the antibody–toxin complexes. Taking into account all these unknowns, 4–8 vials would be theoretically required, given the nominal potency of the Microgen antivenom (2.3 mg V/mL AV) and the amount of venom potentially injected by *V. b. berus* in a severe envenoming (10–18 mg). In Russia, the recommended treatment follows the Besredka’s method [49], which includes an initial in situ subcutaneous administration of 0.1 mL of antivenom in any part of the envenomed patient’s body. If there are no signs of an adverse reaction after 10–15 min, another 0.25 mL is administered, followed by the rest of the vial 15 min later. Once at the hospital and after a clinician has determined the severity of the bite, the patient intramuscularly receives 1–2 vials (mild poisoning) or 4–5 vials if the envenoming was severe. In particularly severe cases, the intravenous administration of the antivenom diluted 1/5–1/10 in saline is recommended. The good consistency between theoretical calculation and clinical practice illustrates the analytical value of combining in vivo neutralization assay and third-generation antivenomics into an integrated platform for the preclinical evaluation of antivenoms.

## 3. Conclusions

Preclinical testing of the antivenom’s efficacy should be performed before the acceptance of a new antivenom. The appropriate evaluation of antivenoms at the preclinical level is necessary to ensure efficacy and safety. Determining the Median Effective Dose (ED_50_) through an in vivo assay provides information about the volume of antivenom (or the venom/antivenom ratio), which is capable of rescuing 50% of the animals tested. In vivo neutralization assays lack toxin-locus resolution as they are not able to identify which toxins are effectively recognized/neutralized and which go unnoticed by the antivenom’s antibodies. Moreover, in almost all existing antivenoms, antibodies toward non-venom antigens represent the most abundant molecules [45,48]. IgGs from normal and hyperimmune sera are indistinguishable chemically and structurally. Furthermore, quantification of the content of anti-toxin antibodies is seldom determined as part of the preclinical evaluation of an antivenom. A deep knowledge of the qualitative and quantitative toxin-recognition landscape is important for eventually improving the clinical profile of antivenoms and for comparing antivenoms from different manufacturers. In this regard, MicroPharm’s monospecific anti-*V. b. berus* ViperaTAb™ antivenom potency is 597 LD_50_s/mL [33]. ViperaTAb™ is an affinity-purified ovine antivenom and therefore, nominally, all its antibodies (20 mg Fab/mL) are specific to *V. b. berus* venom proteins (i.e., its P = 29.85 LD_50_s/mg Fab). In comparison, non-affinity purified Microgen equine antivenom (P = 116 LD_50_s/mL; 120 mg F(ab’)_2_/mL of which 6.24% are anti-toxin antibodies) has a specific potency of 0.97 LD_50_s/mg F(ab’)_2_, which increases to [0.97 × (100/6.24)] = 15.5 LD_50_s/mg F(ab’)_2_ when immunopurified. Thus, based on their toxin-specific antibody combining sites, the Microgen antivenom (two identical antigen-combining sites per F(ab’)_2_ molecule) would have 52% of the lethality neutralization capability of the ViperaTAb™ antivenom (one antigen-combining site per Fab molecule). These data suggest that the horse immunization procedure used to generate the Microgen antivenom was less effective than that applied for immunizing sheep for MicroPharm antivenom, either because of the protocol followed or because of the species of animal used.

Antivenomics represents a powerful complement of and a valuable addition to the in vivo neutralization assay. Consistent with previous third-generation antivenomics reports, our present study highlights our view that the combination of in vivo and in vitro approaches provides a robust toolbox for the evaluation of an antivenom’s preclinical efficacy [35,36,37,50]. Last but not least, our in vivo neutralization/third-generation antivenomics integrated platform represents a significant leap forward towards incorporating the 3Rs (Refinement, Replacement and Reduction of Animals in Research) into the preclinical assessment of snake antivenom efficacy [35].

## 4. Materials and Methods

### 4.1. Venom and Antivenom

Crystallized *V. b. berus* venom (series 04/16, analysis certificate #2CER-04/16, date of approval: 12 May 2016) was obtained from LLC Siberian Serpentarium (Novosibirsk 630007, Russia). Venom used for venomics and antivenomics studies was pooled from an undisclosed exact number (but in the range of 80–100) of adult (over 5 years old; snout to vent length of at least 50 cm) snakes from both sexes (55% males, 45% females), which were collected during the period from April 1 to September 15 from two populations in the Tver and Novosibirsk regions (Russia). Venom was stored at −20 °C until used. Prior to its use for activity measurement, venom was dissolved in 0.12 M NaCl and 0.04 M phosphate at a pH of 7.2 (PBS). Monospecific Anti-Viper Venom Serum (batch number C51, 11.2016, 150AE) used in this study was manufactured by the Federal State Company for Immunobiological Medicines, Microgen (Moscow, Russia) against the same *V. berus* venom pool used in this work and was used within its shelf-life (December 2018).

### 4.2. Animals

Mice with the CD-1 strain were used throughout the study. The protocols involving the use of mice were approved by the Institutional Committee for the Care and Use of Laboratory Animals (CICUA) of the University of Costa Rica (Act 82-2008, date of the approval: 18 September 2008).

### 4.3. Toxic and Enzymatic Venom Activities

#### 4.3.1. Lethality

For the determination of the Median Lethal Dose (LD_50_), groups of five mice (18–20 g) received injections of five venom doses (from 8.9 to 45 µg, in 1.5-fold serial dilutions) in a volume of 0.5 mL of PBS by the intraperitoneal (i.p.) route. Deaths occurring during a period of 48 h were recorded and LD_50_ was estimated by probits [51,52]. For the assessment of neutralization by antivenom, mixtures containing a fixed dose (4 × LD_50_) of venom and five 1.5-fold serial dilutions (from 6.75 to 1.33 mg venom/mL antivenom ratio) were prepared and incubated at 37 °C for 30 min. Aliquots containing 0.5 mL of each mixture were then injected intraperitoneally (i.p.) into groups of five mice (18–20 g). A control group was injected with 4 × LD_50_s of venom incubated with 10 mM sodium phosphate and 135 mM NaCl at a pH of 7.4 (phosphate-buffered saline, PBS) instead of antivenom. Deaths occurring during a period of 48 h were recorded and the neutralizing ability of antivenom was expressed as the Median Effective Dose (ED_50_), i.e., the venom/antivenom ratio at which half of the population of injected mice is protected, which was estimated by probits [51].

#### 4.3.2. Hemorrhagic Activity

The hemorrhagic activity of venom was determined by intradermally injecting five 2.0-fold serial dilutions of venom (from 8 µg to 0.5 µg), which were dissolved in 0.1 mL of PBS, into groups of five mice (18–20 g). Mice were sacrificed by CO_2_ inhalation 2 h after injection. The skin was removed and the diameter of hemorrhagic spots in the inner side of the skin was measured. The Minimum Hemorrhagic Dose (MHD) corresponds to the dose of venom that induced a hemorrhagic spot of 10 mm diameter [53]. For the neutralization experiments, mixtures containing a fixed dose of venom (5 × MHD) and five 1.5-fold serial dilutions (32 to 2 mg venom/mL antivenom ratio) were prepared and incubated at 37 °C for 30 min [52]. After this, aliquots containing 0.1 mL of each mixture were injected intradermally into groups of five 18–20 g mice. A control group of mice was injected with the same dose of venom in PBS. Mice were sacrificed by CO_2_ inhalation 2 h after injection and the area of the hemorrhagic lesion was measured [53]. The antivenom Median Effective Dose (ED_50_) corresponded to the venom/antivenom ratio that reduced the diameter of the hemorrhagic spot by 50% compared to the diameter of the hemorrhagic lesion in mice injected with venom in PBS.

#### 4.3.3. Phospholipase Activity

PLA_2_ activity was determined titrimetrically using egg yolk phospholipids as substrates as previously described [54]. Activity was expressed as µEq fatty acid released per mg protein per min. For the neutralization of PLA_2_ activity, a constant amount of venom was incubated for 30 min at 37 °C, with various dilutions of antivenom. Control tubes included venom and no antivenom. After incubation, PLA_2_ activity was tested as described. The neutralizing ability was expressed as the Median Effective Dose (ED_50_), corresponding to the ratio venom/antivenom at which the PLA_2_ activity of venom was reduced by 50%.

### 4.4. Fractionation and Proteomics Characterization of V. b. berus (Russia) Venom

Two milligrams of crystallized crude venom were dissolved in 200 μL of 5% acetonitrile in water containing 0.1% trifluoroacetic acid (TFA), which was centrifuged to remove debris and separated by reverse-phase HPLC using an LC 1100 High Pressure Gradient System (Agilent Technologies, Santa Clara, CA, USA) and a Teknokroma (Teknokroma, Barcelona, Spain) Europa Protein 300 C18 (0.4 cm × 25 cm, 5 µm particle size, 300 Å pore size) column. The column was developed at a flow-rate of 1 mL/min with a linear gradient of 0.1% TFA in water (solution A) and acetonitrile (solution B) isocratically (5% B) for 5 min, which was followed by 5%–25% B for 10 min, 25%–45% B for 60 min and 45%–70% for 10 min. The eluate was monitored at 215 nm with a reference wavelength of 400 nm. Manually collected fractions were dried in a vacuum centrifuge (Savant™, Thermo Scientific Inc., West Palm Beach, FL, USA), redissolved in water and submitted to quantitative venomics analysis as described in reference [45].

### 4.5. Third-Generation Antivenomics

Third-generation antivenomics [36,37] was applied to assess the immunoreactivity of Russian antivenom against homologous *V. b. berus* venom. Three vials of antivenom (1 mL each, 120 mg/mL) were mixed with 7 mL of MilliQ^®^ water (Burlington, MA, USA), dialyzed against exhaustively MilliQ^®^ water, lyophilized and reconstituted in 10 mL of the coupling buffer (200 mM NaHCO_3_, 500 mM NaCl, pH 8.3). The concentrations of the antivenom stock solution were determined spectrophotometrically using an extinction coefficient for a concentration of 1 mg/mL (ε0.1%) at 280 nm of 1.36 (mg/mL)^−1^ cm^−1^ [55]. Antivenom affinity matrix was prepared in batch as described [45] by incubating 80 mg of Microgen F(ab’)_2_ antivenom with 3 mL of CNBr-activated matrix for 4 h at room temperature. Antivenom coupling yield was 74 mg. Affinity columns containing 10 mg of immobilized antivenom in 405 µL of affinity matrix were washed, equilibrated with 5 volumes of working buffer (PBS, 20 mM phosphate buffer, 135 mM NaCl, pH 7.4) and incubated with increasing amounts (300−1800 μg) of *V. berus* total venom proteins dissolved in ½ matrix volume of PBS. The mixtures were incubated for 1 h at 25 °C in an orbital shaker. For specificity controls, 400 μL of CNBr-activated Sepharose™ 4B matrix without (mock) or with 10 mg of immobilized control (naïve) horse IgGs were incubated with 1200 µg of venom and developed in parallel to the immunoaffinity columns. The non-retained eluates of columns incubated with 300, 600, 900, 1200, 1500 and 1800 μg venom were recovered, respectively, with 5×, 10×, 15×, 20×, 25× and 30× matrix volumes of PBS. The immunocaptured proteins were eluted, respectively, with the same volumes of 0.1 M glycine-HCl, which is a buffer with a pH of 2.7, and brought to neutral pH with 1 M Tris-HCl to a pH of 9.0. To avoid saturation of the downstream reverse-phase chromatographic analysis, aliquots corresponding to 300 µg of initial total venom proteins were concentrated in a Savant SpeedVac™ vacuum system (ThermoFisher Scientific, Waltham, MA, USA) to 40 μL and fractionated by reverse-phase HPLC as above, except that a Discovery^®^ BIO Wide Pore C18 (15 cm × 2.1 mm, 3 μm particle size, 300 Å pore size) column was employed. The column was developed at a flow rate of 0.4 mL/min with a linear gradient of 0.1% TFA in MilliQ^®^ water (solution A) and 0.1% TFA in acetonitrile (solution B), isocratically (5% B) for 1 min, followed by 5–25% B for 5 min, 25–45% B for 35 min and 45–70% B for 5 min and monitored at 215 nm. The toxins recovered in the non-retained (NR) and retained (R) affinity chromatography fractions were quantified using the equation %NRi=100 − [(Ri/(Ri+NRi)) × 100], where Ri corresponds to the area of the same protein “i” in the chromatogram of the fraction retained and eluted from the affinity column [36].

The percentage of antivenom anti-toxin F(ab’)_2_ molecules was calculated by dividing [(1/2 maximal amount (in µM) of total venom proteins bound per anti-*V. berus* vial) × molecular mass (in kDa) of F(ab’)_2_] by the [total amount of F(ab’)_2_ (in mg) per antivenom vial].

The percentage of toxin neutralizing anti-toxin F(ab’)_2_ molecules was calculated by dividing the antivenom’s potency (P) by the maximal amount of total venom proteins bound by mL of antivenom. The potency (P) is the amount of venom (mg) completely neutralized per mL of antivenom. P was calculated using the formula P = [(*n* − 1)/ED_50_] × LD_50_, where “n” is the number of median lethal doses (LD_50_s) that were used as challenge doses to determine the antivenom’s median effective dose, ED_50_. For the calculation of P, LD_50_ and ED_50_ are expressed, respectively, as (mg venom/mouse) and (mL of antivenom that protect 50% of the mice population inoculated with *n* × LD_50_). In the calculation of P, (*n* − 1) × LD_50_ is used instead of the total amount of venom, n × LD_50_, because at the endpoint of the neutralization assay, one LD_50_ is not neutralized and causes the death of 50% of the mice [56,57].

## Figures and Tables

**Table 1:** Lethal, hemorrhagic and PLA_2_ activities of *V. berus berus* venom (V) and neutralization of activities by Russian antivenom (AV) in mice.

Toxic Activity	Activity	Neutralization by Antivenom
Lethality (LD_50_, i.p.)	19.8 (10.7–30.3) ^a^ µg per mouse	ED_50_: 3.1 (1.5–4.7) ^a^ mgV/mL AV
Hemorrhagic (MHD)	2.00 ± 0.79 µg per mouse	8.0 ± 0.1 mgV/mL AV
PLA_2_ Activity	48 ± 20 µeq/mg/min	1.70 ± 0.06 mgV/mL AV

^a^ The 95% confidence limits are expressed in parentheses. Other results are presented as mean ± S.D. (*n* = 5). i.p. intraperitoneal. MHD, minimum hemorrhagic dose.

**Table 2:** Total and concentration-dependent immunoretained (RET) *V. berus berus* (Russia) venom proteins by anti-*V. berus berus* Russian antivenom (10 mg F(ab’)_2_) affinity column. Maximal binding for each RP-HPLC fraction is highlighted in the yellow background.

		*Vipera berus berus* (Russia) Total Venom Proteins (µg)							
RP-HPLC Fraction		100	300	600	900	1200	1500	Toxins in RP-HPLC fraction	
1, 2, 5	µg TOTAL	6.68	20.04	40.08	60.12	80.16	100.2	BPP + KUN	
	µg RET	0.21	0.64	1.10	0.84	0.80	0.64		
3, 4, 6, 7	µg TOTAL	18.97	56.91	113.82	170.73	227.64	288.55	KUN [P00991, P00992]	
	µg RET	3.05	7.08	11.93	17.13	12.30	11.57		
8	µg TOTAL	2.79	8.38	16.75	25.13	33.50	41.88	Dimeric Disintegrin [P0C6A6]	
	µg RET	2.54	7.29	10.13	10.41	10.42	8.46		
9	µg TOTAL	6.60	19.80	39.60	59.40	79.20	99.00	S49-PLA2 [CAE47248]	
	µg RET	6.58	14.96	19.56	19.86	18.09	16.89		
10	µg TOTAL	3.02	9.06	18.12	27.18	36.24	45.30	VEGF [P83942]	
	µg RET	3.02	7.25	9.44	9.87	11.06	9.88		
11–14	µg TOTAL	15.94	31.88	63.76	95.64	127.52	159.40	D49-PLA2 [P31854]	
	µg RET	15.94	33.84	44.21	46.32	49.33	38.33		
16	µg TOTAL	5.43	16.29	32.58	48.87	65.16	81.45	CRISP [ B7FDI1]	
	µg RET	5.42	15.37	15.78	15.92	15.12	14.85		
17–19	µg TOTAL	8.00	24.01	48.01	72.02	96.02	120.03	D49-PLA2 [AAN59990]	
	µg RET	7.98	11.65	15.09	12.01	11.01	10.75		
20, 21	µg TOTAL	1.65	4.96	9.92	14.89	19.85	24.81	SVSP [E5AJX2] + D49-PLA2	
	µg RET	1.65	4.62	5.65	6.22	6.04	5.79		
22	µg TOTAL	9.95	29.84	59.68	89.51	119.35	149.19		
	µg RET	9.39	24.27	28.24	26.77	27.02	24.09		
23	µg TOTAL	0.83	2.49	4.99	7.48	9.97	12.47	PIII-SVMP	
	µg RET	0.83	2.24	2.95	3.18	2.90	2.74		
24	µg TOTAL	0.98	2.94	5.88	8.82	11.76	14.70	SVSP + CTL	
	µg RET	0.87	1.63	5.56	8.31	7.55	7.68		
25–28	µg TOTAL	12.92	38.76	77.52	116.28	155.04	193.8	LAO + PIII-SVMP + CTL	
	µg RET	12.17	36.96	64.92	71.65	82.54	78.98		
29–34	µg TOTAL	4.01	12.03	24.06	36.09	49.02	61.05	LAO + PIII-SVMP + SVSP	
	µg RET	3.78	12.00	22.21	34.78	45.51	49.34		

## Supplementary Materials

The following are available online at http://www.mdpi.com/2072-6651/11/2/90/s1, Table S1: Summary of the MS/MS identification of the SDS-PAGE protein bands of *Vipera berus berus* (Russia).
